# Greener Prescribing: A Quasi-Experimental Study of the Environmental Impact of Metronidazole Dosing Interval Optimization

**DOI:** 10.3390/antibiotics15101035

**Published:** 2026-10-18

**Authors:** Majd Alsoubani, Maureen Campion, Somya Swarup, Pamela Lee, Abarna Pearl, Kap Sum Foong

**Affiliations:** 1 Division of Geographic Medicine and Infectious Diseases, Department of Medicine, Tufts Medical Center, 800 Washington St., Boston, MA 02111, USA; 2 Stuart B. Levy Center for Integrated Management of Antimicrobial Resistance, 136 Harrison Ave, Boston, MA 02111, USA; 3 Department of Medicinne, Tufts University School of Medicine, 145 Harrison Avenue, Boston, MA 02111, USA; 4 Department of Medicine, Harbor-UCLA Medical Center, Torrance, CA 90502, USA; 5 Division of Infectious Diseases, Denver Health Medical Center, Denver, CO 80204, USA

**Keywords:** metronidazole, plastic waste, environmental sustainability, antimicrobial stewardship, planetary health

## Abstract

**Background**: Antimicrobial administration is a major contributor to healthcare-associated waste. We evaluated the environmental impact of changing the default metronidazole dosing frequency from three-times-daily (TID) to twice-daily (BID) administration in a three-hospital system. **Methods**: We conducted a quasi-experimental uncontrolled interrupted time-series study across an integrated health system. Inpatient oral and intravenous metronidazole utilization was assessed from January 2023 through December 2024, before and after a change in the default metronidazole dosing on 1 February 2024. Overall utilization was measured as days of therapy (DOT)/1000 days present (DP), and frequency-specific utilization was standardized as dispensed doses/1000 DP. Plastic waste was estimated using EcoRxChoice, and modeled disposal-related carbon dioxide-equivalent (CO_2_e) emissions reflected facility-specific incineration and transportation processes. **Results:** Overall metronidazole utilization remained similar (36.4 pre-intervention vs. 37.2 DOT/1000 DP post-intervention). After implementation TID utilization decreased relative to baseline (−4.41 dispensed doses/1000 DP per month; 95% CI, −6.02 to −2.80 *p* < 0.001), whereas twice-daily utilization increased (+1.32 dispensed doses/1000 DP per month; 95% CI, 0.37 to 2.27 *p* = 0.007). Mean modeled plastic waste decreased from approximately 200 to 164 kg/month. Over the 11-month post-intervention period, the monthly trend decreased by 14.63 kg/month relative to the pre-intervention trend (95% CI, −21.42 to −7.83; *p* < 0.001) and modeled disposal-related 351.3 kg of CO_2_e. **Conclusions:** Optimizing antibiotic dosing frequency is a simple, scalable antimicrobial stewardship strategy that was associated with reductions in modeled metronidazole plastic waste and modeled disposal-related emissions while leveraging the established clinical non-inferiority of twice-daily metronidazole dosing.

## 1. Background

Antimicrobials are among the most commonly prescribed medications in hospital settings and carry a disproportionate environmental footprint. The environmental impact of antimicrobial use extends beyond the direct effects of pharmaceutical manufacturing and includes contamination of water sources through pharmaceutical residues and downstream ecological effects that may contribute to the selection and spread of antimicrobial resistance [1]. In addition, antimicrobial administration relies on single-use products such as medication containers, syringes, tubing, saline bags, gloves, and packaging with each administration. These materials further augment healthcare-associated waste and carbon emissions throughout their production, transportation, and disposal [2]. Given the high volume of antimicrobial use in hospitals, even relatively small reductions in the environmental footprint of individual doses could translate into meaningful reductions in healthcare-associated waste when implemented at scale.

Antimicrobial stewardship programs have traditionally focused on improving clinical outcomes, reducing unnecessary antimicrobial exposure, limiting adverse events and antimicrobial resistance, and controlling costs [3]. More recently, however, environmental sustainability has emerged as an additional dimension of responsible antimicrobial prescribing. This has become particularly relevant from a One Health perspective, which recognizes the interconnected health of humans, animals, and the environment [4]. Incorporating sustainability into antimicrobial stewardship provides an opportunity to align high-quality patient care with efforts to reduce the environmental consequences of healthcare delivery.

Most published environmental stewardship interventions have focused on discontinuing unnecessary therapy or transitioning treatment from intravenous to oral formulations [5,6]. Recent work evaluating the transition from intravenous to oral metronidazole has demonstrated meaningful environmental benefits, with substantial reductions in carbon dioxide emissions [5]. These findings underscore the potential environmental benefits of optimizing antibiotic administration practices, particularly when such changes can be implemented without compromising clinical outcomes [7].

Although route optimization is an important sustainability strategy, environmental benefit could be achieved by reducing the number of doses administered per day. Each medication administration represents a discrete healthcare activity requiring staff time and, depending on the route of administration, multiple disposable supplies. Reducing the number of doses administered per day may therefore decrease resource consumption and waste even when the antimicrobial agent, route, and overall duration of therapy remain unchanged. Metronidazole is an antibiotic commonly used for anaerobic coverage [8]. Traditionally, it has been administered three times daily (TID). However, emerging evidence indicates that twice-daily (BID) dosing is clinically non-inferior to TID dosing, with comparable outcomes in mortality, clinical cure, and re-admission [7,9]. Prior studies have suggested that reduced-frequency dosing may also improve adherence, reduce adverse drug effects, and decrease nursing workload [9]. Importantly, because BID dosing requires fewer administration events per day than TID dosing, it reduces the number of single-use supplies consumed per treatment course independent of the total quantity of drug delivered although the magnitude varies by route of administration. This represents a potential mechanism by which dosing-frequency optimization can reduce plastic waste.

At Tufts Medicine, pharmacy services implemented a system-wide intervention to preferentially use BID metronidazole dosing for all adult patients, excluding those with *Clostridioides difficile* or central nervous system infections because evidence supporting reduced-frequency dosing for these indications is limited. Despite growing evidence supporting BID dosing, the environmental impact of such dosing optimization has not been characterized. This study aimed to evaluate the environmental impact of a pharmacy-led intervention to transition from TID to BID metronidazole dosing in hospitalized adult patients.

## 2. Results

Overall metronidazole utilization was similar over time. Both combined mean oral and intravenous metronidazole use was 36.4 DOT/1000 DP pre-intervention and 37.2 DOT/1000 DP post-intervention. Mean intravenous metronidazole utilization was 29.06 DOT/1000 DP pre-intervention and 29.40 DOT/1000 DP post-intervention, while mean oral utilization was 7.31 and 7.79 DOT/1000 DP, respectively. Intravenous therapy accounted for 79.9% of total metronidazole DOT before the intervention and 79.1% after the intervention, indicating minimal change in the distribution of oral versus intravenous therapy.

### 2.1. Changes in Metronidazole Utilization

Before the implementation of the intervention, TID utilization was rising significantly (1.77 dispensed doses/1000 DP per month; *p* < 0.001) while BID utilization remained stable (0.36 dispensed doses/1000 DP per month; *p* = 0.227).

**Figure 1:**
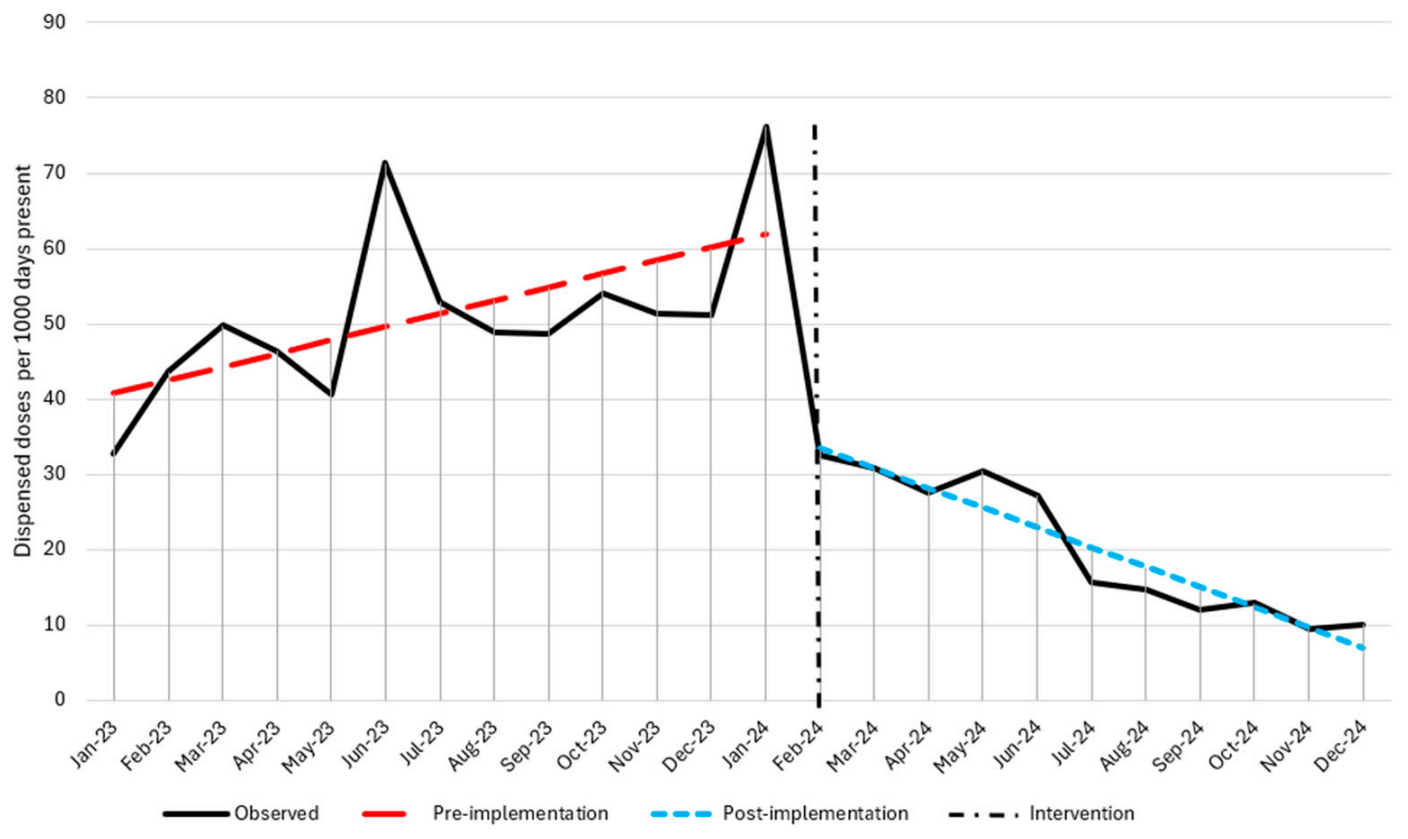
Monthly defined daily dose of three-times-a-day metronidazole before and after implementation.

Implementation was associated with an immediate level decrease of 25.83 dispensed doses/1000 DP (95% CI, −36.96 to −14.69; *p* < 0.0001). In addition, the monthly trend decreased by 4.41 dispensed doses/1000 DP in TID utilization relative to the baseline trend (95% CI, −6.02 to −2.80; *p* < 0.0001) (Figure 1, Table 1).

**Figure 2:**
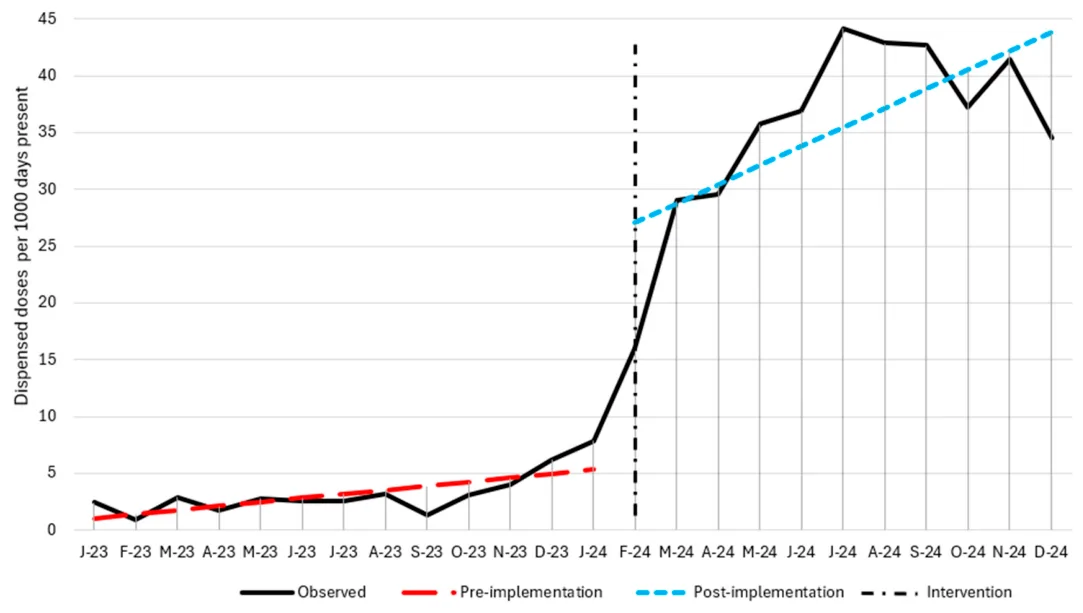
Monthly defined daily dose of twice-a-day metronidazole before and after implementation.

A corresponding increase in BID utilization was observed. Implementation was associated with an immediate level increase of 20.06 dispensed doses/1000 DP (95% CI, 13.49 to 26.62; *p* < 0.0001). The monthly trend subsequently increased by an additional 1.32 dispensed doses/1000 DP relative to the baseline trend (95% CI, 0.37 to 2.27; *p* = 0.007) (Figure 2, Table 2).

### 2.2. Plastic Waste Outcomes and Environmental Impact

Mean metronidazole-associated plastic waste decreased from approximately 200 kg/month (SD 51.6) pre-intervention to approximately 164 kg/month (SD 24.7) post-intervention.

**Figure 3:**
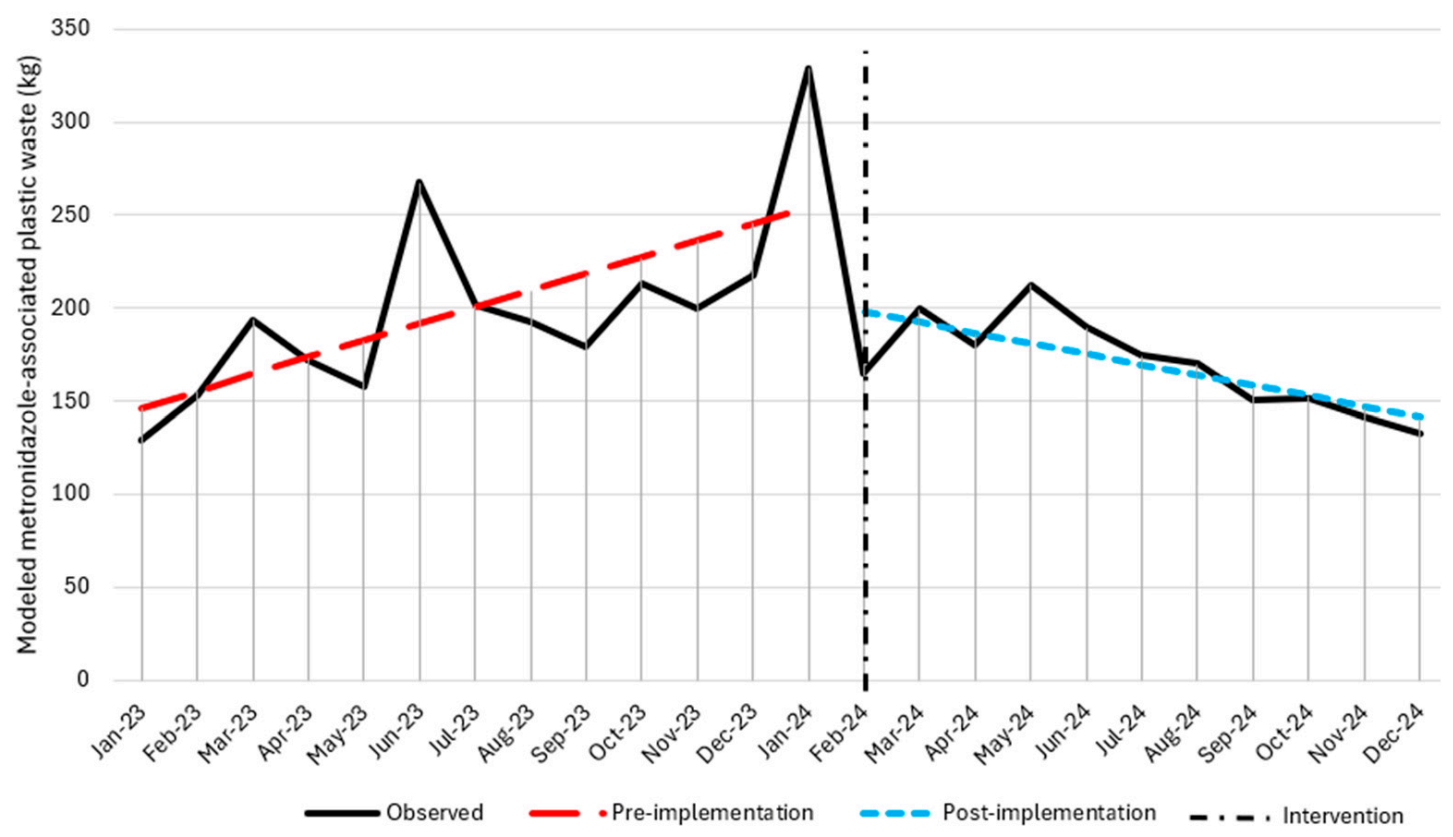
Monthly modeled plastic waste associated with metronidazole use before and after implementation.

Before implementation, plastic waste generation increased by 9.00 kg/month (95% CI, 4.83 to 13.18; *p* < 0.0001). ITS analysis showed a significant immediate post-intervention reduction in plastic waste generation of 51.03 kg (95% CI, −97.88 to −4.17; *p* = 0.033). The monthly trend subsequently decreased by −14.62 kg/month (95% CI, −21.42 to −7.83; *p* < 0.0001) (Table 3; Figure 3). Separately from the ITS analysis, the dispensing-based environmental calculation demonstrated a cumulative reduction of approximately 130 kg of modeled metronidazole-associated plastic waste over the 11-month post-intervention period. Detailed environmental-model inputs and calculations are provided in Supplementary Tables S1 and S2.

Based on the modeled transportation and disposal processes, this reduction corresponded to an annual estimated 351.3 kg CO_2_e reduction in disposal-related greenhouse-gas emissions.

In the exploratory chart review of 50 patients receiving BID metronidazole after implementation, no documented infection recurrence or escalation from BID to TID dosing was identified during the reviewed follow-up period. Given the small sample size and descriptive nature of this assessment, these findings should not be interpreted as evidence of clinical safety or non-inferiority.

## 3. Discussion

Following implementation of the dosing-frequency intervention, the shift toward BID metronidazole dispensing was associated with lower modeled metronidazole-associated plastic waste. Total metronidazole utilization remained relatively similar while dosing frequency shifted to BID dosing.

These findings add to a growing body of literature demonstrating that relatively modest changes in antimicrobial prescribing and administration can produce meaningful environmental benefits [5,6,10,11,12,13]. Although our study only looked at disposal waste, others have evaluated broader carbon footprint estimates. A previous initiative focusing on promoting oral rather than intravenous metronidazole reported a 64% reduction in intravenous metronidazole use, correlating with an estimated annual savings of 35.1 metric tons of CO_2_e [5]. In another study, switching to highly bioavailable oral alternatives to avoid unnecessary intravenous antibiotic therapy produced an estimated annual savings of 0.35 metric tons of CO2e and approximately 2000 kg of total waste [6]. A cross-sectional antimicrobial stewardship study likewise estimated that accepting recommendations to discontinue intravenous therapy or to transition to oral treatment avoided 261.2 kg of CO_2_e and 106.5 kg of medical waste [10]. Similarly, early transition to oral therapy in uncomplicated *Enterobacterales* bloodstream infections was not associated with increased mortality or relapse and was associated with an estimated reduction of 38.8 kg of CO_2_e, along with financial savings [11]. Lastly, prior work comparing TID meropenem to once-daily ertapenem illustrated reductions in vials, saline bags, and tubing for disposal [2].

Our findings highlight an additional, often under-recognized benefit of antimicrobial stewardship, the potential to reduce healthcare-associated waste and carbon emissions in addition to its established clinical effects [9]. This intervention is notable for its simplicity and scalability. Following implementation of the system-wide CPOE default change, aggregate dispensing patterns shifted toward BID dosing and were associated with lower plastic waste without restricting or altering antibiotic selection. The reduction is consistent with fewer scheduled and dispensed doses, which would be expected to reduce the single-use supplies required for medication administration.

Framing antimicrobial use within a One Health perspective, which incorporates environmental outcomes alongside patient-centered and economic outcomes, provides a more comprehensive assessment of antimicrobial stewardship interventions [4,14]. More broadly, environmentally sustainable antimicrobial prescribing should not be viewed solely as a strategy to reduce healthcare-associated waste. Antimicrobial prescribing contributes to pharmaceutical residues entering the environment, resource consumption associated with medication production and administration, and greenhouse-gas emissions generated throughout healthcare delivery. These environmental consequences can in turn have a broader implication for human and population health [15].

Antimicrobial stewardship programs are uniquely positioned to identify prescribing interventions that simultaneously maintain clinical effectiveness and reduce resource consumption and environmental impact. Tools such as EcoRxChoice, which offers published methods for estimating plastic waste associated with antimicrobial administration, may facilitate inclusion of environmental metrics in stewardship evaluations [16]. Although the reduction associated with an individual treatment course may be modest, metronidazole is widely used across acute care settings and the cumulative effect of eliminating one administration per patient per day may be meaningful when implemented across multiple hospitals.

In addition to environmental benefits, reducing dosing frequency may improve operational efficiency. A TID schedule requires more administration events, increasing nursing workload, and the potential for delays or administration errors [2]. Twice-daily dosing may also improve patient adherence and reduce gastrointestinal and neurologic side effects based on the current available evidence [17].

This study has several limitations. First, it was conducted within a single healthcare system using aggregate data, which may limit generalizability. Second, plastic waste was derived from a validated, published calculator rather than direct measurement and thus represents modeled, rather than observed, reductions. Because EcoRxChoice is designed to underestimate waste, the true reduction may be larger [16]. Third, CO_2_e emissions were modeled for the disposal phase only and do not reflect the full product life cycle. The transportation-emissions assumptions represent an illustrative calculation based on fixed assumptions regarding transportation distance, this estimate carries additional uncertainty not captured in the reported figures, actual emissions may differ depending on local waste management practices, and this estimate should not be interpreted as directly measured or definitively avoided emissions. Fourth, the short study duration precluded formal seasonal adjustment. The residual autocorrelation in the BID model and unequal residual variance between pre- and post-intervention periods in the TID and BID models may affect the precision of those estimates, the findings should be considered exploratory and interpreted primarily in terms of the direction and magnitude of observed changes rather than definitive statistical or causal effects. Fifth, we were unable to determine the specific clinical reasons clinicians selected TID rather than BID dosing or canceled doses after they were dispensed due to the retrospective study design. Sixth, because utilization data were aggregated across the health system, hospital-specific prescribing patterns and differences in intervention uptake could not be assessed, and we could not exclude the influence of concurrent stewardship interventions or confirm individual dose administration or reliably characterize doses that were subsequently held, missed, canceled, or returned. Seventh, aggregate dispensing data should not be interpreted as a direct measure of prescriber compliance or intervention fidelity. Finally, clinical outcomes were not assessed in this analysis for all patients. The exploratory chart review was not designed to systematically assess clinical outcomes or establish the safety or non-inferiority of BID dosing. However, prior studies have consistently demonstrated the clinical non-inferiority of BID metronidazole dosing [7,17].

## 4. Methods

### 4.1. Study Design and Setting

We conducted a quasi-experimental interrupted time-series (ITS) study at Tufts Medicine, a 1095-bed integrated healthcare system comprising one academic tertiary medical center (415 beds) and two community-based hospitals (445 and 234 beds). The intervention was implemented on 1 February 2024, resulting in a 13-month pre-intervention period and an 11-month post-intervention period. This was an uncontrolled ITS design as no unaffected comparator antibiotic was available to serve as a control series.

Aggregate inpatient oral and intravenous metronidazole usage data were obtained from the pharmacy dispensing system from 1 January 2023 through 31 December 2024. Days present (DP) for each month were extracted from the National Healthcare Safety Network Antibiotic Use and Resistance database. DP were used to standardize antimicrobial utilization over time and account for changes in inpatient census.

Overall metronidazole utilization, irrespective of dosing frequency, was assessed as days of therapy (DOT)/1000 DP. DOT was defined as the aggregate number of days on which a patient received at least one dose of metronidazole. Because DOT does not distinguish between dosing frequencies administered on the same treatment day, it could not be used to evaluate changes specifically attributable to the transition from TID to BID dosing. Therefore, frequency-specific metronidazole utilization was assessed.

The pharmacy dataset included prescribed order frequency and the number of doses dispensed per day, which allowed metronidazole utilization to be classified as BID or TID. Frequency-specific dispended doses were therefore derived from metronidazole quantities associated with orders classified as BID or TID, rather than inferred from total metronidazole quantity alone. Monthly frequency-specific dispensed doses were then calculated and standardized per 1000 DP (dispensed doses/1000 DP).

### 4.2. Environmental Impact Assessment

Plastic waste associated with metronidazole administration was estimated using EcoRxChoice, a publicly available web-based calculator funded by the Society of Infectious Diseases Pharmacists (accessed March, 2026) [16]. This tool incorporates medication formulation, preparation requirements, and administration supplies to estimate waste generated per dose. We entered the oral and intravenous metronidazole regimens dispensed during the study period to obtain plastic waste per dose, which was multiplied by monthly dispensing volumes to estimate total monthly plastic waste. The estimated plastic waste was 2.4 g per oral dose and 119.2 g per intravenous dose.

We modeled facility-specific disposal processes, including incineration at an external facility located approximately 668 miles away and transportation via a gasoline-powered vehicle, using emission factors from the 2025 U.S. Environmental Protection Agency’s Emission Factors Hub (Supplementary Table S3) with methods similar to Hofmeister et al. [18].

The specific vehicle class, trip structure (one-way versus round-trip), load-sharing across a mixed waste stream, and allocation of transportation emissions to metronidazole-associated waste specifically were not independently tracked beyond travel distance and fuel type and represent a source of uncertainty in the disposal-phase estimate.

### 4.3. Intervention and Outcomes

The intervention was implemented on 1 February 2024 by changing the default metronidazole dosing frequency in the computer order entry system (CPOE) from TID to BID. February 2024 was included in the post-intervention period. The intervention applied to metronidazole use across the three hospitals within the health system. At implementation, a system-wide email was sent to all clinicians outlining the change in default metronidazole dosing frequency and specifying the clinical indications for which TID dosing should be used. Providers could still select TID dosing for any indication.

The prespecified primary outcomes were the monthly change in TID and BID metronidazole utilization (dispensed doses/1000 DP), comparing pre- and post-intervention periods. The secondary outcome was the monthly change in metronidazole-associated plastic waste expressed in kilograms.

Overall metronidazole DOT/1000 DP was evaluated to assess whether metronidazole use remained similar between the two periods. As a balancing measure, a random sample of 50 hospitalized patients who received BID metronidazole following implementation underwent retrospective chart review. Eligible patients included all hospitalized adult patients receiving BID metronidazole dosing and were followed for 1 month post completion of treatment. Medical records were reviewed by authors (MA and MC) for infection recurrence and escalation of metronidazole therapy from BID to TID dosing. This descriptive review was intended to identify potential clinical concerns following implementation and was not designed or powered to establish clinical safety or non-inferiority.

### 4.4. Statistical Analysis

We summarized monthly outcomes using means and standard deviations (SDs). We performed an uncontrolled ITS analysis using segmented linear regression to evaluate level and trend changes following the intervention, fit separately for BID utilization, TID utilization (dispensed doses/1000 DP), and plastic waste (kilograms) using the equation Y_t_ = β_0_ + β_1_(Time_t_) + β_2_(Intervention_t_) + β_3_(Post_Time_t_) + ε_t_, where Time is a continuous month counter, Intervention is a pre/post indicator, and Post_Time counts months since intervention (0 before, incrementing after). β_1_ is the pre-intervention trend, β_2_ the immediate level change, and β_3_ the post-intervention trend change. February 2024, the month the CPOE default changed, was coded as the first post-intervention month (Intervention = 1, Post_Time = 1) rather than excluded as a transition period, since the change was implemented directly at the order-entry level. Primary models were fit using SPSS GENLIN with a normal distribution and identity link. Residual diagnostics were obtained using the corresponding regression-based procedures in SPSS.

Durbin–Watson statistics showed no meaningful autocorrelation for the TID (2.17) and plastic-waste (2.33) models. The BID model (1.37) showed mild autocorrelation, noted as a limitation. Heteroscedasticity was assessed using Levene’s test comparing residual variance between pre- and post-intervention periods. Variance differed significantly for the TID (*p* = 0.049) and BID (*p* = 0.003) models but not the plastic-waste model (*p* = 0.147). Seasonality was not modeled given the 24-month series length. Residual diagnostic (Shapiro–Wilk) showed non-normality across models, driven by several outlying months. Effect estimates are reported with 95% confidence intervals (CI) accordingly. A two-sided *p* value < 0.05 was considered statistically significant. The interrupted time-series analysis was used to evaluate changes in the level and monthly trend of modeled plastic waste following implementation. The plastic-waste reduction was calculated separately from the observed monthly dispensing data and was not derived from the ITS coefficients.

All statistical analyses were performed using SPSS, version 25 (IBM, Armonk, NY, USA). This study was reviewed by the Tufts Medical Center Institutional Review Board which deemed it exempt from full review.

## 5. Conclusions

Evidence-based optimization of antibiotic dosing frequency can serve as a potential practical, low-cost, and scalable antimicrobial stewardship strategy. In this uncontrolled interrupted time-series analysis, implementation of a BID metronidazole default was associated with reductions in modeled healthcare-associated plastic waste and modeled disposal-related CO_2_e emissions, while leveraging existing evidence supporting comparable clinical effectiveness of BID metronidazole dosing.

## Figures and Tables

**Table 1:** Uncontrolled interrupted time-series estimates of changes in three-times-daily metronidazole utilization following dosing interval optimization.

	Parameter Estimate (95% CI)	*p* Value
Intercept pre-intervention, dispensed doses/1000 DP	38.98 (31.12, 46.85)	<0.0001
Pre-intervention segment slope dispensed doses/1000 DP per month	1.77 (0.78, 2.76)	<0.0001
Level change post-intervention dispensed doses/1000 DP	−25.83 (−36.96, −14.69)	<0.0001
Change in slope post-intervention dispensed doses/1000 DP per month	−4.41 (−6.02, −2.80)	<0.0001

Abbreviation: CI, confidence interval; DP, days present. The pre-intervention slope represents the monthly change in utilization before implementation. The level change represents the estimated difference in utilization immediately following implementation compared with the expected level based on the pre-intervention trend. The change in slope represents the difference between the post-intervention and pre-intervention monthly trends.

**Table 2:** Uncontrolled interrupted time-series estimates of changes in twice-daily metronidazole utilization following dosing interval optimization.

	Parameter Estimate (95% CI)	*p* Value
Intercept pre-intervention, dispensed doses/1000 DP	0.67 (−3.97, 5.31)	0.778
Pre-intervention segment slope dispensed doses/1000 DP per month	0.36 (−0.22, 0.95)	0.227
Level change post-intervention dispensed doses/1000 DP	20.06 (13.49, 26.62)	<0.0001
Change in slope post-intervention dispensed doses/1000 DP per month	1.32 (0.37, 2.27)	0.007

Abbreviation: CI, confidence interval; DP, days present. The pre-intervention slope represents the monthly change in utilization before implementation. The level change represents the estimated difference in utilization immediately following implementation compared with the expected level based on the pre-intervention trend. The change in slope represents the difference between the post-intervention and pre-intervention monthly trends.

**Table 3:** Uncontrolled interrupted time-series estimates of changes in total plastic waste (kilogram) associated with metronidazole use following dosing interval optimization.

	Parameter Estimate (95% CI)	*p* Value
Intercept pre-intervention, kg	137.56 (104.45, 170.69)	<0.0001
Pre-intervention segment slope, kg/month	9.00 (4.83, 13.16)	<0.0001
Level change post-intervention, kg	−51.03 (−97.88, −4.17)	0.033
Change in slope post-intervention, kg/month	−14.63 (−21.42, −7.89)	<−0.0001

Abbreviation: CI, confidence interval; kg, kilogram. The pre-intervention slope represents the monthly change in modeled plastic waste before implementation. The level change represents the estimated difference in modeled plastic waste immediately following implementation compared with the expected level based on the pre-intervention trend. The change in slope represents the difference between the post-intervention and pre-intervention monthly trends.

## Data Availability

Data is available upon request from the corresponding author.

## Supplementary Materials

The following supporting information can be downloaded at https://www.mdpi.com/article/10.3390/antibiotics15101035/s1, Table S1: Monthly intravenous metronidazole utilization and modeled plastic waste by dosing frequency; Table S2: Monthly oral metronidazole utilization and modeled plastic waste by dosing frequency; Table S3: Greenhouse Gas Emissions Calculations for 130 kg Plastic Waste (Transportation included). References [19,20,21,22] are cited in the supplementary materials.
