# Supplementary material for: Greener Prescribing: A Quasi-Experimental Study of the Environmental Impact of Metronidazole Dosing Interval Optimization
Source: Antibiotics (Basel). 2026 Oct 18;15(10):1035. doi: 10.3390/antibiotics15101035 (PMC13700826; doi:10.3390/antibiotics15101035)
Supplement: Supplementary file 1 [file antibiotics-15-01035-s001.zip › antibiotics-4526778-supplementary.pdf]

antibiotics

MDPI

Table S1. Monthly intravenous metronidazole utilization and modeled plastic waste by dosing frequency.

	Three-Times-Daily Dosing		Twice Dosing		Total Days of Therapy/1000 Days Present
	Days of therapy	Plastic Waste (output from EcoRxChoice)	Days of therapy	Plastic Waste (output from EcoRxChoice)	
1/31/2023	462	1483.00	10	97901.20	17.69
2/28/2023	595	185.40	1	126064.00	24.33
3/31/2023	696	1165.20	8	147447.60	26.58
4/30/2023	625	2198.00	15	132446.00	25.06
5/31/2023	585	2330.4	16	123972.00	22.83
6/30/2023	1078	2198.00	15	228400.80	42.17
7/31/2023	696	768.00	5	147447.60	26.86
8/31/2023	700	1483.00	10	148295.00	26.62
9/30/2023	683	900.40	6	144706.80	26.88
10/31/2023	790	2780.6	19	167388.0	30.13
11/30/2023	717	5243.4	36	151936.20	28.72
12/31/2023	828	7891.6	54	175411.80	31.71
1/31/2024	1288	12552.40	86	272862.80	48.16
2/29/2024	452	33976.20	233	95756.20	25.54
3/31/2024	500	56512.2	388	105925.00	31.90
4/30/2024	376	56247.40	386	79655.60	28.69
5/31/2024	440	76638.40	526	93214.00	35.38
6/30/2024	355	68164.20	468	75220.00	32.20
7/31/2024	246	91786.00	630	52141.60	32.56
8/31/2024	213	89720.40	616	45163.80	30.44
9/30/2024	177	78518.60	539	37537.20	27.73
10/31/2024	221	78068.40	536	46858.60	27.21
11/30/2024	125	83894.40	576	26521.00	26.48
12/31/2024	171	77221.00	530	36239.60	25.24

Table S2. Monthly oral metronidazole utilization and modeled plastic waste by dosing frequency.

	**Three-Times-Daily Dosing**		**Twice Times Daily Dosing**		**Total Days of Therapy/1000 Days Present**
	Days of therapy	Plastic Waste (output from EcoRxChoice)	Days of therapy	Plastic Waste (output from EcoRxChoice)	
1/31/2023	119	25223.40	33	4846.20	5.70
2/28/2023	117	24826.20	13	1933.20	5.31
3/31/2023	183	38781.80	43	6276.20	8.53
4/30/2023	166	35193.60	15	2198.00	7.09
5/31/2023	128	27116.80	33	4846.20	6.12
6/30/2023	154	32651.40	29	4263.60	7.06
7/31/2023	225	47706.00	39	5693.60	10.12
8/31/2023	169	35842.40	47	6858.80	8.10
9/30/2023	148	31353.80	16	2330.40	6.40
10/31/2023	177	37537.20	36	5243.40	7.93
11/30/2023	180	38133.00	34	4978.60	8.16
12/31/2023	121	25673.60	61	8924.40	6.54
1/31/2024	162	34346.20	64	9321.60	7.92
2/29/2024	129	27368.40	54	7891.60	6.82
3/31/2024	71	15054.60	151	22006.40	7.97
4/30/2024	112	23727.20	138	20126.20	9.41

5/31/2024	115	24376.00	125	18246.00	8.79
6/30/2024	108	22879.80	161	23489.40	10.52
7/31/2024	33	7030.80	163	23754.20	7.28
8/31/2024	54	11466.40	163	23754.20	7.97
9/30/2024	28	5931.80	196	28547.40	8.68
10/31/2024	18	3839.80	154	22456.60	6.18
11/30/2024	42	8924.2	155	22589.00	7.44
12/31/2024	17	3641.20	110	16048.00	4.57

Table S3. Greenhouse Gas Emissions Calculations for 130kg Plastic Waste (Transportation included).

Source of Greenhouse Gas Emissions	Description	Value
**CO_2 Emissions From Transportation**	Distance From Hospital to Incineration (roundtrip in miles)[a]	1336
	Truck miles per gallon [1]	7.00
	Fuel Type Emission Factor (kg CO_2/gallon) [2]	8.78
	Assumed number of truck trips	1
	Truck Capacity (kilograms) [3]	13607.80
	Attributable Emissions %	0.96%
	Total Transport Emissions (kg CO_2)	16.01
**CO_2 Emissions From Incineration**	Emissions from Incineration [4]	335.32
	Total Incineration Emissions (kg CO_2)	*335.32*
	**Total (kg CO_{2e})**	**351.33**

a-Distances are estimated for services from Boston, MA to Grafton, OH

Transportation Emissions = Attributable Emissions % = (Mass of Plastic Waste/Truck Capacity)

Transportation Emissions for One Truck of Waste = x Fuel type Emission Factor

CO_2 Emissions from Transportation for 130kg of Waste = Attributable Emissions % x Transportation Emissions for One Truck of Waste

CO_2 Emissions From Incineration = Mass of Plastic Waste over Study Period in Tons x Emissions from Combustion

References

1. Sandhu, G.S.; Frey, H.C.; Bartelt-Hunt, S.; Jones, E. In-use activity, fuel use, and emissions of heavy-duty diesel roll-off refuse trucks. *J. Air Waste Manag. Assoc.* **2015**, *65*, 306-323. https://doi.org/10.1080/10962247.2014.990587.
2. GHG Emission Factors Hub. Available online: https://www.epa.gov/climateleadership/ghg-emission-factors-hub (accessed on 8 March 2026).
3. Cargo Estimates. Available online: https://bigtruckrental.com/.
4. Emission Factors for Greenhouse Gas Inventories. Available online: https://www.epa.gov/system/files/documents/2025-01/ghg-emission-factors-hub-2025.pdf (accessed on 8 March 2026).

Disclaimer/Publisher's Note: The statements, opinions and data contained in all publications are solely those of the individual author(s) and contributor(s) and not of MDPI and/or the editor(s). MDPI and/or the editor(s) disclaim responsibility for any injury to people or property resulting from any ideas, methods, instructions or products referred to in the content.
